# CTCF and Rad21 Act as Host Cell Restriction Factors for Kaposi's Sarcoma-Associated Herpesvirus (KSHV) Lytic Replication by Modulating Viral Gene Transcription

**DOI:** 10.1371/journal.ppat.1003880

**Published:** 2014-01-09

**Authors:** Da-Jiang Li, Dinesh Verma, Tim Mosbruger, Sankar Swaminathan

**Affiliations:** 1 Division of Infectious Diseases, Department of Internal Medicine, University of Utah School of Medicine, Salt Lake City, Utah, United States of America; 2 Division of Infectious Diseases, Department of Internal Medicine, University of Utah School of Medicine, Salt Lake City, Utah, United States of America; 3 Huntsman Cancer Institute, University of Utah School of Medicine, Salt Lake City, Utah, United States of America; 4 Division of Infectious Diseases, Department of Internal Medicine, University of Utah School of Medicine, Salt Lake City, Utah, United States of America; 5 George E. Wahlen Department of Veterans Affairs Medical Center, Salt Lake City, Utah, United States of America; Baylor College of Medicine, United States of America

## Abstract

Kaposi's sarcoma-associated herpesvirus (KSHV) is a human herpesvirus that causes Kaposi's sarcoma and is associated with the development of lymphoproliferative diseases. KSHV reactivation from latency and virion production is dependent on efficient transcription of over eighty lytic cycle genes and viral DNA replication. CTCF and cohesin, cellular proteins that cooperatively regulate gene expression and mediate long-range DNA interactions, have been shown to bind at specific sites in herpesvirus genomes. CTCF and cohesin regulate KSHV gene expression during latency and may also control lytic reactivation, although their role in lytic gene expression remains incompletely characterized. Here, we analyze the dynamic changes in CTCF and cohesin binding that occur during the process of KSHV viral reactivation and virion production by high resolution chromatin immunoprecipitation and deep sequencing (ChIP-Seq) and show that both proteins dissociate from viral genomes in kinetically and spatially distinct patterns. By utilizing siRNAs to specifically deplete CTCF and Rad21, a cohesin component, we demonstrate that both proteins are potent restriction factors for KSHV replication, with cohesin knockdown leading to hundred-fold increases in viral yield. High-throughput RNA sequencing was used to characterize the transcriptional effects of CTCF and cohesin depletion, and demonstrated that both proteins have complex and global effects on KSHV lytic transcription. Specifically, both proteins act as positive factors for viral transcription initially but subsequently inhibit KSHV lytic transcription, such that their net effect is to limit KSHV RNA accumulation. Cohesin is a more potent inhibitor of KSHV transcription than CTCF but both proteins are also required for efficient transcription of a subset of KSHV genes. These data reveal novel effects of CTCF and cohesin on transcription from a relatively small genome that resemble their effects on the cellular genome by acting as gene-specific activators of some promoters, but differ in acting as global negative regulators of transcription.

## Author Summary

Kaposi's sarcoma-associated herpesvirus (KSHV) is a human virus that causes Kaposi's sarcoma and lymphoma. KSHV establishes a lifelong infection in B lymphocytes, and persists in a latent form as circular DNA molecules. Reactivation and replication yield infectious virions, allowing transmission and maintenance of latent infection. The cellular mechanisms controlling reactivation remain incompletely characterized. Host proteins that regulate RNA transcription play an important role in controlling viral reactivation. In this study, we used high-throughput techniques to analyze the binding of two cellular proteins, CTCF and Rad21, to the KSHV genome as the virus reactivated to produce infectious virions. We found that these proteins dissociate from the latent genome when reactivation occurs. We also found that depleting cells of these proteins increases virus production as much as a hundredfold. Depleting the cell of CTCF or Rad21 caused complex changes in the synthesis of RNAs by KSHV, with the amounts of most KSHV RNAs increasing greatly. We also showed that Rad21 and CTCF are needed for the virus to synthesize RNAs efficiently. Our study provides new insights into how the cell uses CTCF and Rad21 to limit KSHV's ability to synthesize RNA and reactivate from latency to produce infectious virus.

## Introduction

Infection with Kaposi's sarcoma-associated herpesvirus (KSHV, HHV8) is causally associated with Kaposi's sarcoma (KS), primary effusion lymphoma (PEL) and multicentric Castleman's disease (for a review, see reference [1]). KSHV maintains a persistent latent infection as an episome in B lymphocytes, from which it occasionally reactivates, enters a lytic cycle of replication, and produces infectious virions. Released virions infect other lymphocytes to maintain the latent reservoir or are transmitted from person-to-person in saliva. Cell-mediated immunity is essential for limiting KSHV reactivation and pathogenesis, but cellular epigenetic regulatory mechanisms may also play an important role in limiting viral replication. The balance between lytic and latent infection is an important determinant of pathogenicity.

Lytic herpesvirus reactivation, while often more common in states of immunosuppression, is nevertheless apparently stochastic, and may occur quite variably among fully immunocompetent individuals [2]. Lytic replication and viral gene expression are important in pathogenesis for several reasons. First, expansion of the reservoir of infected cells is at least partly dependent on recurrent reactivation of human gammaherpesviruses. Thus long-term acyclovir suppression of lytic replication led to a significant decrease over time in the latent Epstein-Barr virus (EBV) load in B lymphocytes of immunocompetent patients [3]. Second, lytic replication and gene expression appears to contribute to oncogenesis in several settings where even a minority of infected cells is permissive for lytic replication [4]–[6]. Several lytic KSHV gene products have anti-apoptotic, proliferative or immunosuppressive properties, increasing the likelihood of malignant transformation by paracrine and autocrine mechanisms [7], [8]. The role of lytic replication in oncogenesis is supported by the decreased incidence of KS in KSHV infected individuals who received long-term antiviral therapy for other infections [9]. Understanding the basic mechanisms by which the host cell maintains control of lytic viral replication and viral strategies to overcome such control is therefore central to devising novel therapies aimed at these control points.

Host proteins that play multiple roles in chromatin organization, transcriptional regulation and chromosome segregation have recently been shown to also bind herpesvirus genomes at specific sites and regulate gene expression [10]–[13]. CTCF is an 11 zinc finger sequence-specific DNA binding protein with roles in transcription activation and repression, gene insulation, enhancer blocking and long range chromatin interactions [14], [15]. CTCF binds to between 14,000 to 20,000 sites in the human genome and is functionally important in regulation of several hundred genes based on knockdown studies [16]–[18]. Initial studies suggested that CTCF exerted activating or repressing effects on promoters by direct binding in the manner of classic transcription factors [19]. However, its role in global gene regulatory functions was demonstrated by its ability to block enhancer function when interposed between enhancer elements and target promoters [20]. Subsequent studies have shown that CTCF binding mediates insulation throughout the human genome [18], [21]. In addition, CTCF may act as a barrier element, demarcating regions of heterochromatin and open chromatin, thereby isolating areas of low and high transcriptional activity. Based on binding studies delineating intra-chromosomal interactions, CTCF mediates three dimensional chromatin structure via long-range interactions.

Cohesin, a complex of four proteins, SMC1, SMC3, SCC1/Rad21 and SA1/2, essential for chromatid segregation, has also been recognized as a global regulator of transcription (for a review, see reference [22]). The four proteins form a ring-shaped structure that encloses chromatids. Several other proteins are associated with cohesin, and regulate the dynamic association of cohesin with chromatin as it is sequentially loaded and dissociated from chromosomes during mitosis and segregation. Specificity of cohesin localization is complex and likely mediated by multiple proteins including NIPBL, mediator, transcription factors and CTCF. Thus while cohesin binds to many CTCF sites, it also binds to sites on the genome independently of CTCF. Although cohesin may have both positive and negative effects on transcription, many of its effects are thought to be mediated by facilitating and stabilizing long-range interactions between promoters and enhancers to which it binds. The most likely mechanism is that cohesin causes topological linking of DNA sequences *in cis* similar to its role in chromatid linkage *in trans*. Cohesin also is involved in regulation of polII pausing at promoters and relieves pausing, promoting RNA elongation [23].

CTCF and cohesin bind at distinct sites on herpesvirus genomes, including herpes simplex virus, EBV and KSHV. CTCF has been implicated in regulating gene expression during latent EBV infection by mechanisms that likely involve both insulator function and modification of genome conformation by causing formation of intragenomic loops [13]. During KSHV infection in primary effusion lymphoma cells, both cohesin and CTCF play a regulatory role in latent and possibly lytic gene expression [12], [24]–[26]. Chromosome conformation capture assays have demonstrated that a cohesin/CTCF site in the 5′ region of the major latency KSHV transcript forms contacts with a site close to the primary gene necessary for lytic reactivation (ORF50/RTA) and with the 3′ region of the latency region. [12]. Mutation of the CTCF site led to increased latency gene expression, suggesting that CTCF and cohesin play a repressive role in latent gene expression. Interestingly however, deletion of this site also led to a loss of stable viral episome maintenance.

Although knockdown of cohesin components led to increased transcription of lytic genes in PEL cells, depletion of CTCF had virtually no such effect [24]. Conversely, mutation of the CTCF site, which would be predicted to disrupt both cohesin and CTCF binding, led to decreased lytic gene expression. In this study, we have performed a detailed analysis of the role of cohesin and CTCF in regulating KSHV lytic replication. By employing siRNAs specific for CTCF and cohesin, we have explored their role in regulating KSHV lytic replication. Using ChIP-Seq, we have defined at high resolution the dynamic changes in cohesin and CTCF binding that occur during lytic KSHV replication and reactivation from latency. The distinct regulatory roles of cohesin and CTCF have also been further defined by transcriptional profiling of infected cells undergoing lytic replication under conditions of cohesin and CTCF depletion. These studies reveal novel mechanisms of gene regulation by CTCF and cohesin during KSHV replication and establish their role as host restriction factors for KSHV replication.

## Materials and Methods

### Cells and plasmids

293 and 293T cells were grown at 37°C in Dulbecco's modified Eagle's medium (DMEM) supplemented with 10% fetal bovine serum (FBS) and glutamine. iSLK cells [27] were maintained in DMEM containing 10% charcoal stripped FBS (Sigma) and glutamine with 250 µg/ml neomycin and 1 µg/ml puromycin. iSLK cells were infected with WT KSHV derived from bacmid BAC16, expressing eGFP and hygromycin resistance [28]. KSHV-infected iSLK cells were maintained in 1.2 mg/ml hygromycin, 250 µg/ml neomycin and 1 µg/ml puromycin.

### Immunoblotting analysis

Protein samples were analyzed by sodium dodecyl sulfate-polyacrylamide gel electrophoresis (SDS-PAGE) and immunoblotted with rabbit polyclonal anti-CTCF (Millipore), anti-Rad21 (Bethyl) or anti-actin monoclonal antibody (Sigma) and horseradish peroxidase-conjugated secondary antibody (GE Healthcare), followed by visualization with a Clarity Western ECL Substrate Kit (Bio-Rad). Image capture was performed with a BioRad GelDoc system.

### Luciferase assays

293T cells were plated at 600,000/well in 6-well plates. CTCF or Rad21 were knocked down by transfection with On-target SMARTpool CTCF siRNA or Rad21 siRNA, or mock-depleted with negative control siRNA (see below). 48 h later, 293T cells were transfected with 1 ug/well pDD398 (ORF57 promoter-luciferase reporter) plus 1 ug/well pDD267 (ORF50 expression plasmid in pCDNA3) or empty pCDNA3 vector, using Transit-293 (Mirus) per the manufacturer's protocol. Each transfection was performed in triplicate. 48 h later, cells were harvested and lysed in reporter lysis buffer (Promega). Luciferase assays were performed in triplicate with 0.5 ul of each lysate using Promega's Luciferase Reporter Assay System per the manufacturer's protocol.

### CTCF and Rad21 knockdown

CTCF (L-020165-00-0005), Rad21 (L-006832-00-0005) and negative control On-target plus Smart Pool siRNAs (D-001810-03) were purchased from Thermo Scientific. Each siRNA was transfected into SLK KSHV WT cells using Lipofectamine RNAiMAX (Invitrogen) according to the manufacturer's protocol and a 10 nM final concentration of siRNA. Similar experiments were also performed with siGENOME Non-Targeting SiRNA #5, D-001210-05-05, SiGENOME human CTCF siRNA, M-020165-02-0005 and SiGENOME human Rad21 siRNA, M-006832-01-0005, purchased from the same manufacturer. Immunoblotting was performed to verify knockdown of the relevant protein.

### ChIP assays

The chromatin immunoprecipitation (ChIP) assay was performed as follows. Briefly, 25 million iSLK cells were harvested and washed with cold PBS containing protease inhibitors (Sigma). Protease inhibitors were added to all solutions in this protocol with the exception of low salt wash buffer. Cells were transferred to DNA LoBind tubes (Eppendorf) in 1 ml of PBS. Cell fixation was performed by addition of 37% formaldehyde to 1% final concentration and rocking gently for 10 min at room temperature. 2M glycine was added to 0.128M final concentration. After centrifugation and washing with cold PBS, cell pellets were resuspended in 2.5 ml ice cold swelling buffer (5 mM PIPES pH 8.0, 85 mM KCl, 0.5% NP-40) for 10 min on ice. Cell nuclei were pelleted and resuspended in 2 ml SDS lysis buffer (1% SDS, 10 mM EDTA and 50 mM Tris-HCl, pH 8). Lysed nuclei were sonicated on ice to yield approximately 500-bp DNA fragments using a Branson Sonifier 450. The extent of DNA fragmentation was confirmed by gel electrophoresis of aliquots of the sonicated nuclear preparation. After extract clearing by centrifugation, supernatants were diluted 1∶5 in CHIP dilution buffer (0.01% SDS, 1% Triton X-100, 1.2 mM EDTA, 16.7 mM Tris-HCL, pH 8 and 167 mM NaCl). Rabbit polyclonal IgG (Bethyl) 1 µg/ml and 50 µl/ml of 50% Protein-A agarose slurry were used to preclear supernatants for 2 hour. Protein-A beads were pelleted, and supernatants were used for immunoprecipitation. 2% of each supernatant was reserved for use as input samples. 8 ug anti-CTCF (Millipore) or Rad21 (Bethyl) antibody was added and the tubes were rocked at 4°C overnight. 30 µl/2 ml of 50% Protein-A agarose slurry was added and incubated for 2 hours at 4°C with rotation. The tubes were centrifuged rapidly and the beads were washed 3 times with cold low salt wash buffer (0.1% SDS, 1% Triton X-100, 2 mM EDTA, 20 mM Tris-HCl, pH 8.1 and 150 mM NaCl) and once with cold TE buffer. Antibody-protein complexes were eluted 6 times with freshly prepared, pre-heated elution buffer (1% SDS, 0.84% NaHCO3) at 65°C. Total elution volume was 1 ml for each immunoprecipitation. Sodium chloride was added to the elutions and input samples to a final concentration of 200 mM NaCl and heated at 65°C for 4 hours. RNase A and proteinase K were added to digest RNA and protein. Finally, DNA was purified from the eluted samples using Qiaquick PCR Purification Kit (Qiagen) according to the manufacturer's protocol.

After DNA purification, libraries were constructed from the chromatin-immunoprecipitated DNA and input samples using the ChIP-Seq DNA sample prep kit (Illumina, San Diego, CA). Single-end reads of 50 cycles were sequenced on an Illumina HiSeq2000 platform. Sequence reads were mapped to the KSHV genome (NC_009333.1). Library preparations, Illumina sequencing and sequencing data analysis were performed by the University of Utah Huntsman Cancer Institute Microarray facility.

### RNA isolation and analysis

Total cellular RNA was isolated from washed cell pellets using Qiazol and Qiagen miRNeasy columns according to the manufacturer's protocols. mRNA was purified from 6 µg total RNA using Qiagen Oligotex mRNA Midikit (Qiagen). cDNA libraries were prepared using the ABI high Capacity cDNA Reverse Transcription Kit with RNase inhibitor (Applied Biosystems). Real-time Quantitative PCR (qPCR) was performed with SYBR green PCR Master Mix (Applied Biosystems) according to the manufacturer's protocol. Each sample was analyzed in triplicate with gene specific primers and β-actin was used as the endogenous control. The gene-specific primers were as follows:

ORF6-2093F: 5′-CTGCCATAGGAGGGATGTTTG-3′;

ORF6-2158R: 5′- CCATGAGCATTGCTCTGGCT-3′

ORF25-3733F:5′-CTCGGCGACGTGCTATACAAT-3′;

ORF25-3803R: 5′-TGCCGACAAGGACTGTACATG-3′;

ORF47 Q1F: 5′-AGCCTCTACCCTGCCGTTGTTCT-3′;

ORF47 Q1R 5′-ACGACCGCGACTAAAAATGACCT-3′;

ORF57 Q1-5: 5′-GCAGAACAACACGGGGCGGA-3′

ORF57Q2-3′:5′-GTCGTCGAAGCGGGGGCTCT-3′

ORF59 Q1F, 5′-CTCCCTCGGCAGACACAGAT-3′;

ORF59 Q1R, 5′-GCGTGGTGCACACCGACGCCC-3′;

K2-430F: 5′-ACCCTTGCAGATGCCGG-3′;

K2-494R: 5′- GGATGCTATGGGTGATCGATG-3′

K5 Q1F: 5′-TAAGCACTTGGCTAACAGTGT-3′

K5 Q1R: 5′-GGCCACAGGTTAAGGCGACT-3′

vIRF-1_lytF: 5′-CGGCATAGCTGTGCTTACCA-3′;

vIRF-1R: 5′- CATTGTCCCGCAACCAGACT-3′;

PAN Q1F, 5′-CCGCCGATTGTGGGTTGATT-3′;

PAN Q1R, 5′-TTTTGTTCTGCGGGCTTATGGAG-3′;

B-actin Q1F: 5′-TCAAGATCATTGCTCCTCCTGAG-3′

B-actin Q1R: 5′-ACATCTGCTGGAAGGTGGACA-3′

### High-throughput deep sequencing of RNA

RNA samples from iSLK cells were prepared using Qiagen miRNeasy kits. 1.5 µg of each RNA were poly(A) selected, and libraries were prepared using the Illumina TruSeq RNA sample preparation protocol (catalog no. RS-930-2001) and validated using an Agilent Bioanalyzer. RNA sequencing libraries were sequenced (50 cycle single-end reads) using an Illumina HiSeq2000 instrument.

### Induction of lytic gene expression, virus replication and quantification of infectious virus release and virus replication

To induce KSHV lytic gene expression or virus replication. iSLK cells were treated with 1 ug/ml doxycycline. Cells were harvested at 24 or 48 hr for RNA preparation. For virus production, supernatants of the cells were harvested 5 days after induction, cleared by centrifugation twice, and filtered through a 0.80 µM pore-size cellulose acetate filter. Serial dilutions of supernatants were used to infect 293T cells. 48 hours after infection, flow cytometry was performed on samples in which 20∼40% of the infected cells were GFP positive. Based on the dilution factor, virus titers in the iSLK cell supernatant were calculated. Pellets of the cells from which supernatant was harvested were processed for DNA isolation using Qiagen DNeasy Blood and Tissue kit. 50 ng of each DNA were used for qPCR using primers specific for ORF59 (see above) and SYBR green PCR MasterMix (ABI).

### Bioinformatic analysis of RNA-Seq and ChIP-Seq data

#### Reference generation

Reference fasta files were generated by combining the standard chromosome sequences from hg19 and the KSHV sequence from NCBI reference NC_009333.1. Ensembl transcript annotations for hg19 were downloaded from the UCSC table browser and combined with the KSHV gene annotations listed in NCBI reference NC_009333.1. Gene annotations were created by merging transcripts with the same gene identifier. All possible splice junction sequences from each gene's transcripts were generated using USeq's MakeTranscriptome application using a radius of 46. These splice junction sequences were added to the combined hg19 and KSHV sequences and run through Novoindex (v2.8) to create the RNASeq reference index. The ChIP-Seq reference index was created by running Novoindex on the combined hg19 and KSHV sequences using default settings.

#### RNASeq

Reads were aligned to the transcriptome reference index described above using Novoalign (v2.08.01), allowing up to 50 alignments for each read. USeq's SamTranscriptomeParser application was used to select the best alignment for each read and convert the coordinates of reads aligning to splices back to genomic space.

Differential gene expression was measured using USeq's Defined Region Differential Seq application. Briefly, the numbers of reads aligned to each gene annotation were calculated. The counts were then used in DESeq, which normalizes the signal and determines differential expression [29]. Fold change values of KSHV were not variance stabilized.

#### ChIP-Seq

Reads were aligned to the ChIP-seq reference index described above using Novoalign (v2.08.01). Duplicate alignments were removed from hg19, but not KSHV. USeq's Sam2SUSeq program was used to generate coverage tracks [30]. Sam2SUSeq generates per base read depth stair-step graph files for genome browser visualization. By default, values were scaled per million mapped KSHV reads with no score thresholding. IGV v2.3 was used to visualize and analyze coverage tracks [31]. Peaks were called using USeq's ChipSeq application, using a window size of 250 bp and a peak shift of 150 bp. The peaks called at each time point were then merged. The number of alignments overlapping each merged peak were calculated for the input and IP fractions at all three time points. These raw hg19 and KSHV peak counts were scaled by multiplying by M/N, where N is the total aligned reads in the sample being scaled and M is the total aligned reads in the input day 0 sample. The KSHV peak counts were additionally scaled by multiplying by P/Q, where P is the number of reads that align to KHSV in the input day 0 sample and Q is the number of reads aligned to KHSV, scaled by M/N, in the input day T sample, where T is the time point of the sample being scaled. This second normalization step was done to account for increasing KHSV reads over time. Once the reads were normalized, log_2_ fold-changes and binomial p-values were calculated.

## Results

### CTCF depletion enhances lytic KSHV replication and alters KSHV gene expression

**Figure 1:**
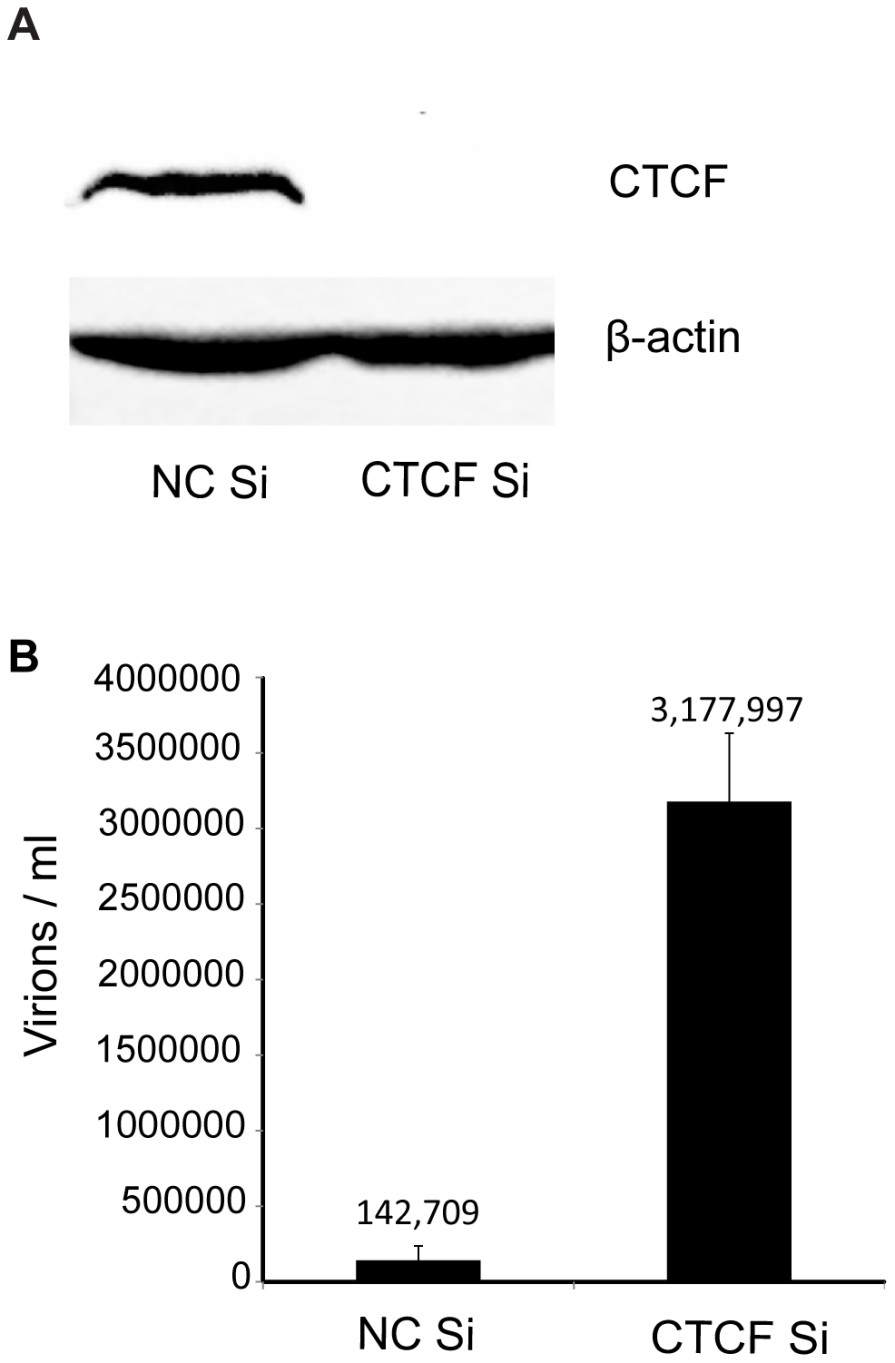
Effect of CTCF depletion on KSHV virion production. A. CTCF knockdown in iSLK cells. Cells were transfected with either negative control siRNA (NC Si) or siRNA specific for CTCF (CTCF Si). Cell lysates were prepared 48 h after transfection and analyzed by immunoblotting with an anti-CTCF antibody (upper panel). Blots were stripped and re-probed with an anti-actin antibody as a loading control (lower panel). B. KSHV virus production in cells depleted of CTCF. KSHV-infected iSLK cells were transfected with either control siRNA (NC Si) or CTCF-specific siRNA (CTCF Si) and KSHV replication was induced by treatment with doxycycline. Supernatants from induced cells were used to infect 293 cells. Virus passage was quantitated by flow cytometry of GFP-positive 293 cells. Each transfection/induction was performed in triplicate and three replicate infections were performed with each supernatant. Uninduced cell supernatants from control and CTCF depleted cells contained less than twenty-five detectable infectious particles per ml (data not shown).

In order to investigate the potential role of CTCF as a host restriction factor for KSHV lytic replication and reactivation from latency, we specifically depleted KSHV infected cells of CTCF prior to inducing lytic replication. Robust and synchronous reactivation of KSHV from latency was achieved by using SLK cells stably transduced with a doxycycline-inducible viral transactivator, KSHV ORF50/RTA [27]. These RTA-inducible SLK cells (iSLK) were infected with the Bac16 KSHV strain that expresses hygromycin resistance and GFP [28]. Infected cells were 100% GFP positive when maintained under hygromycin selection (data not shown). Highly efficient CTCF depletion was achieved by lipid-mediated transfection of iSLK cells with siRNA specific for CTCF (Figure 1A). In order to assess the effect of CTCF depletion on KSHV reactivation and virion production, cells were transfected with either CTCF-specific siRNA or control siRNA and treated with doxycycline 48 hours later. KSHV reactivation was allowed to proceed and virion-containing supernatant was harvested 120 hours after induction of lytic replication with doxycycline. Infectious virus production was measured by infection of 293 cells with serial dilutions of virus supernatant followed by flow cytometry of infected cells. Virus titer in the supernatant can thus be accurately quantitated as GFP-transducing units. As shown in Figure 1B, CTCF depletion prior to induction of lytic replication led to a marked increase in virion production (20–25 fold), compared to control cells induced to replicate. There was no visible or flow cytometry-detectable release of virus without doxycycline-induced RTA expression from either CTCF depleted cells or in control cells, indicating that RTA is still absolutely required for lytic replication (data not shown). Previous investigations of the role of CTCF in KSHV replication in PEL cells detected no effect of CTCF knockdown on KSHV lytic replication [24]. This is therefore the first demonstration of CTCF acting as a restriction factor for KSHV virus production.

**Figure 2:**
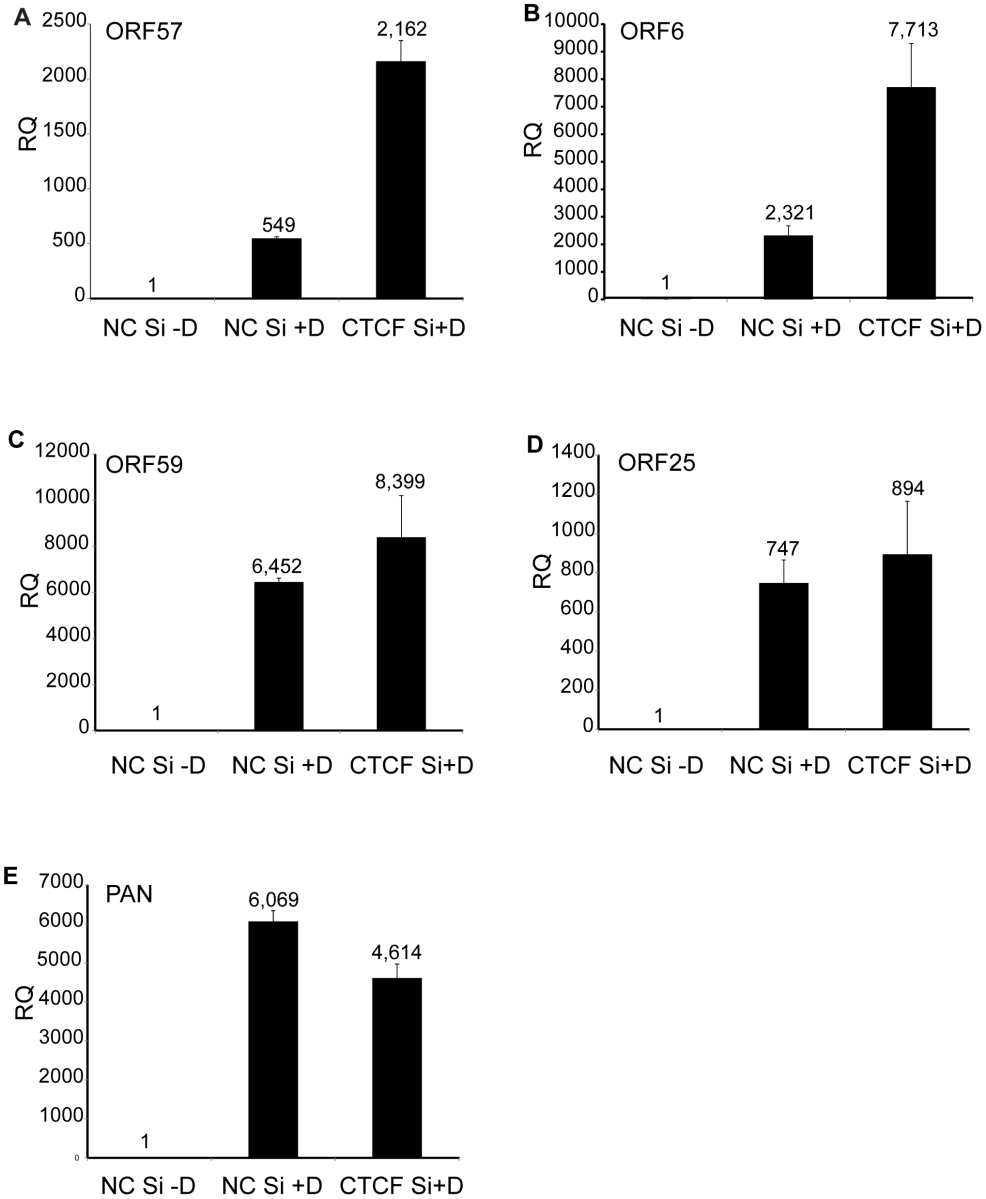
Effect of CTCF depletion on KSHV lytic cycle gene expression. KSHV-iSLK cells were depleted of CTCF (CTCF Si) or mock-depleted (NC Si) by siRNA transfection followed by treatment with doxycycline (−D, mock treatment; +D, doxycycline treatment) to induce lytic replication. RNA was prepared 48 h after induction of replication and relative quantification of mRNA expression (RQ) for each lytic gene was determined by qPCR. Results for ORF57 (A), ORF6 (B), ORF59 (C), ORF25 (D) and PAN (E) are shown. Each transfection was performed in triplicate and qPCR was performed with three technical replicates per sample. The level of expression for each RNA was normalized to the level of expression in uninduced cells.

CTCF may act as a transcriptional activator or inhibitor by a variety of mechanisms, including alteration of chromosomal conformation by formation of intrachromosomal loops. Previous studies have reported decreased transcription of several lytic KSHV genes upon partial CTCF knockdown, indicating CTCF-mediated transcriptional activation [12], [24]. Our experiments suggested that CTCF might also repress KSHV lytic genes, leading to increased virus production when CTCF was completely depleted. In order to determine if the increased KSHV replication observed when CTCF was knocked down in SLK cells might be due to transcriptional mechanisms, we assessed changes in mRNA levels of representative KSHV lytic genes by qPCR after CTCF knockdown. Cells were transfected with CTCF or control siRNA and cellular RNA was isolated 48 h after lytic replication was induced as previously described. While ORF57 (early) and ORF6 (early) lytic mRNA expression were enhanced approximately four-fold by CTCF depletion, there was a less significant increase in other early (ORF59) or late (ORF25) lytic mRNAs (Figure 2 A–D). In addition, expression of PAN RNA, a nuclear non-coding polyadenylated RNA important for lytic reactivation [32], was not enhanced by CTCF depletion (Figure 2E). It therefore appeared that CTCF knockdown might enhance expression of KSHV genes in a gene-specific manner, consistent with transcriptional repression due to site-specific binding.

### Dynamic changes in CTCF binding to the KSHV genome during virus reactivation

**Figure 3:**
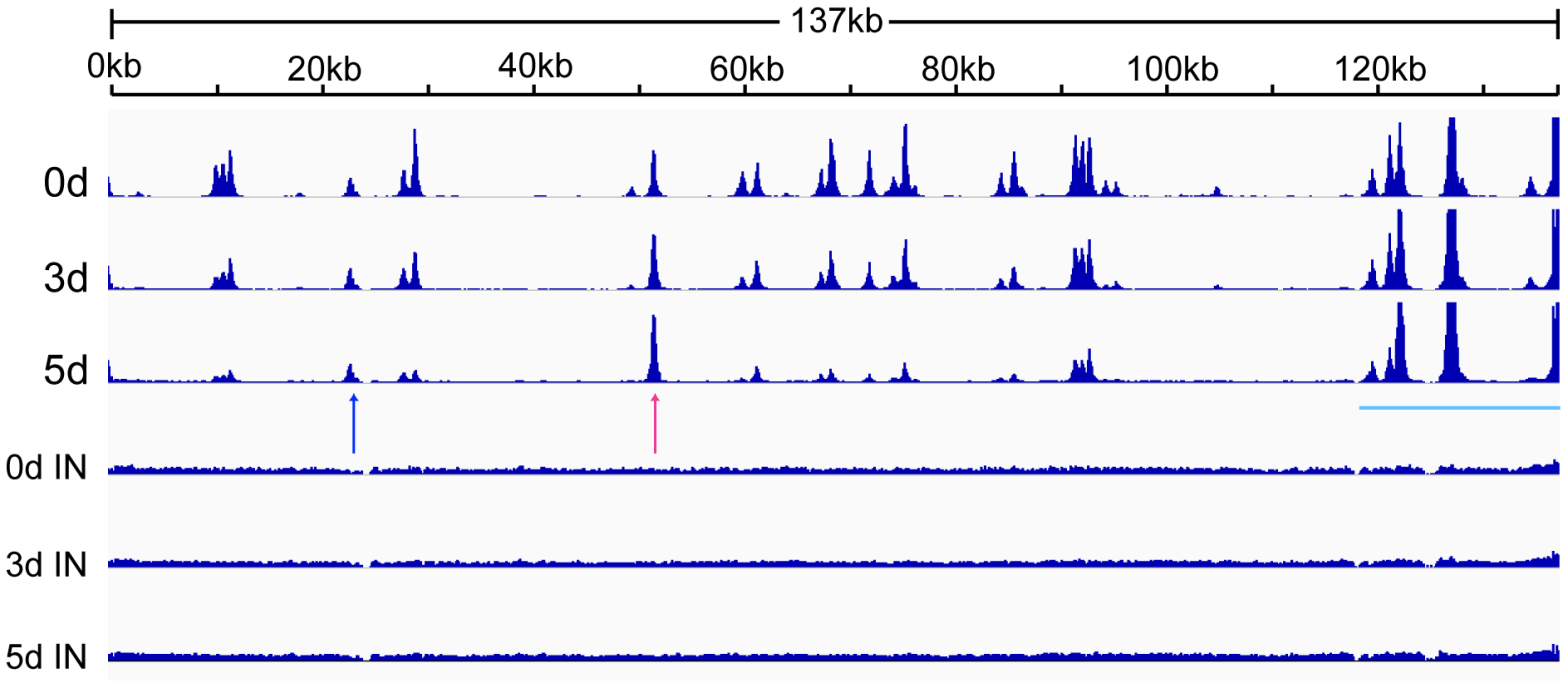
Changes in CTCF binding to the KSHV genome during KSHV reactivation and lytic replication. ChIP-SEQ assays were performed on cell samples obtained at 0, 3, and 5 d after induction of lytic KSHV replication in iSLK cells. Relative read numbers are plotted on the vertical axis versus the reference KSHV genome map on the horizontal axis. CTCF ChIP results for each time point are shown on the upper three panels and the corresponding input samples (IN) are shown on the lower three panels. The tracks depict coverage per base, scaled per million mapped KSHV reads. The region including the latency locus where CTCF binding is maintained is shown by a blue bar, and two additional sites of retained CTCF binding are shown by red and blue arrows (see text).

CTCF binds at specific sites on the KSHV genome during latency and mediates intrachromosomal interactions, primarily between the ORF50 region and the major latency region [12], [24], [25]. Since CTCF appeared to play a role in restricting productive KSHV replication, it seemed likely that CTCF dissociation from one or more sites might occur upon lytic reactivation. A comprehensive analysis of dynamic changes in CTCF binding during reactivation from latency has not been previously performed. We therefore performed ChIP-Seq studies on iSLK cells at serial times after induction of lytic replication to characterize CTCF binding to the KSHV genome during the process of reactivation from latency. It should be noted that the system employed in these studies does not require sodium butyrate or other chemical inducers, which have broad effects on gene expression and epigenetic state. Rather, induction of lytic KSHV replication in iSLK cells relies solely on transcriptional activation by KSHV RTA. After treatment with doxycycline to induce RTA expression and lytic replication, we harvested cells at 0, 3 and 5 days after induction. Cells were treated with formaldehyde to cross-link DNA and protein, followed by DNA fragmentation and immunoprecipitation with anti-CTCF antibody. Immunoprecipitated DNA and input DNA were then analyzed by high-throughput DNA sequencing. The results, shown in Figure 3, reveal several important aspects of dynamic CTCF changes during KSHV reactivation. First, the high resolution map provided by the deep sequencing identifies at least thirty distinct areas of CTCF localization during latency. All contain high-probability sequence motifs and are consistent with previously published literature [24], [26], [33]. Second, there are clearly broad regions in which CTCF binding decreases as lytic replication progresses. Importantly, however, wholesale eviction of CTCF from the genome does not occur. Rather, binding at most sites in the latency gene locus, from approximately nt 117,000 to the 3′ end of the genome remains preserved (blue bar). Similarly, binding at the major CTCF site at approximately nt 52,000 (red arrow) is also maintained. Further evidence of the site-specific nature of these dynamic changes in CTCF binding is evident at another site (blue arrow) where CTCF occupancy is maintained despite its loss at neighboring sites.

### Rad21 depletion enhances KSHV virion production

**Figure 4:**
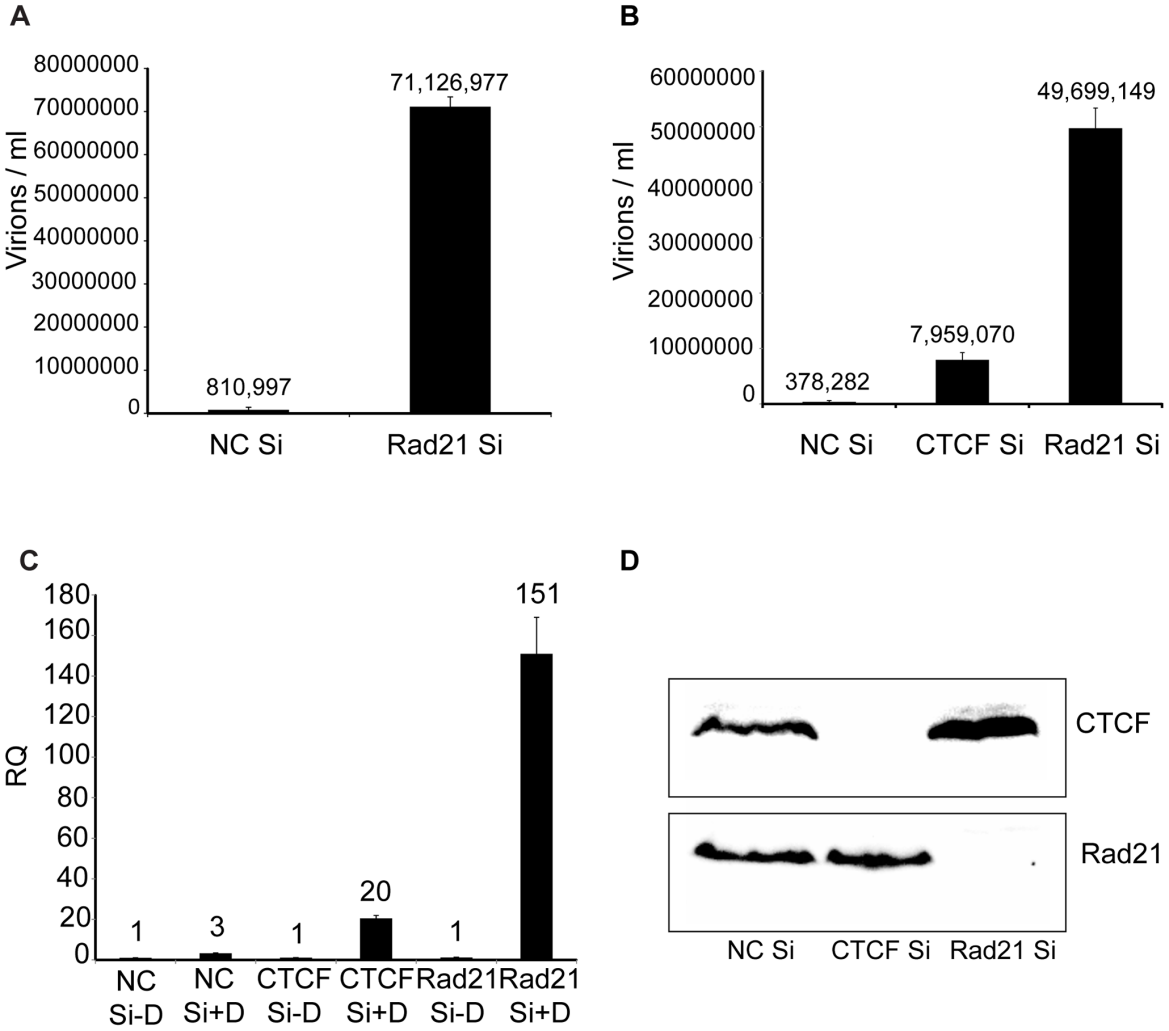
KSHV virus production in cells depleted of Rad21. A. KSHV-infected iSLK cells were transfected with either control siRNA (NC Si) or Rad21-specific siRNA (Rad21 Si), and KSHV replication was induced by treatment with doxycycline. Supernatants from induced cells were used to infect 293 cells. Virus passage was quantitated by flow cytometry of GFP-positive 293 cells. Each transfection/induction was performed in triplicate and three replicate infections were performed with each supernatant. B. CTCF knockdown (CTCF Si) and Rad21 knockdown (Rad21 Si) were performed on iSLK cells in parallel with control siRNA transfection (NC Si). Lytic replication was induced and virus production was measured by passage of virus to KSHV-negative 293 cells as in (A) above. C. CTCF, Rad21 and negative control knockdown were performed in iSLK cells as in (B) above and DNA was isolated from cell pellets. KSHV genome copy number was measured by qPCR. Cells were either untreated (−D) or treated with doxycycline (+D) to induce replication. RQ (relative quantitation). D. Immunoblotting of lysates from cells used in virus production experiments in panel B above was performed with anti-CTCF and anti-Rad21 antibodies to verify completeness of CTCF and Rad21 depletion. Lysates were prepared from cells harvested at the time of replication induction with doxycycline.

Previous work in lymphoma cells had indicated that cohesin components, including Rad21, but not CTCF, repress KSHV immediate-early gene expression [24]. Since CTCF depletion alone led to greatly increased KSHV virion production, and cohesin is known to bind to many CTCF sites, it was of interest to determine the effect of cohesin disruption on KSHV replication in our system. Cohesin is a complex of four core proteins, SMC1, SMC3, SCC1/Rad21 and SA1/2 that encloses chromatids and may act to facilitate intrachromosomal looping. Depletion of Rad21 effectively disrupts cohesin function in DNA binding and transcriptional regulation [34]. Rad21 knockdown was therefore carried out with Rad21-specific siRNA, and KSHV virion production after induction of replication was measured, as was done in CTCF knockdown experiments (Figure 4). Virus production after Rad21 depletion was compared to virus production in cells transfected with control siRNA and revealed that Rad21 depletion enhanced KSHV virion yield even more robustly than CTCF depletion (approximately 90-fold, Figure 4A). In subsequent experiments, CTCF depletion was performed in parallel with Rad21 depletion and confirmed that Rad21 represses KSHV virion production more efficiently than does CTCF (Rad21 depletion enhanced virus production approximately 130-fold versus 20-fold for CTCF depletion, Figure 4B).

To determine whether the effect of Rad21 or CTCF KD on infectious KSHV virion production was due to increased KSHV replication, we measured KSHV genome copy number by qPCR on DNA samples from cells that were induced to replicate after KD of either CTCF or Rad21. The results demonstrated that the KSHV copy number in each sample correlated extremely well with the increases in infectious virion titer. KD of CTCF or Rad21 led to approximately 20-fold or 150-fold increases in copy number, respectively (Figure 4C). Completeness of Rad21 and CTCF depletion was verified by Western blotting of lysates from siRNA-transfected cells (Figure 4D).

These experiments were repeated with a completely different pool of siRNAs and a different control siRNA. The results were consistent with those shown above (Figure S1). In order to ensure that the effects of siRNA depletion were not due to adventitious effects of siRNA carryover during infection, we performed infections of 293 cells with virus-containing supernatant and added supernatant from siRNA-transfected but uninduced cells, which had no effect on virus titers measured by flow cytometry (Figure S2). These data demonstrate that Rad21, although it is thought to bind primarily at CTCF sites, has effects independent of CTCF binding, and is an even more potent repressor of KSHV replication.

### Rad21 binds in a discrete pattern distinct from CTCF and undergoes dynamic changes in localization during lytic replication

**Figure 5:**
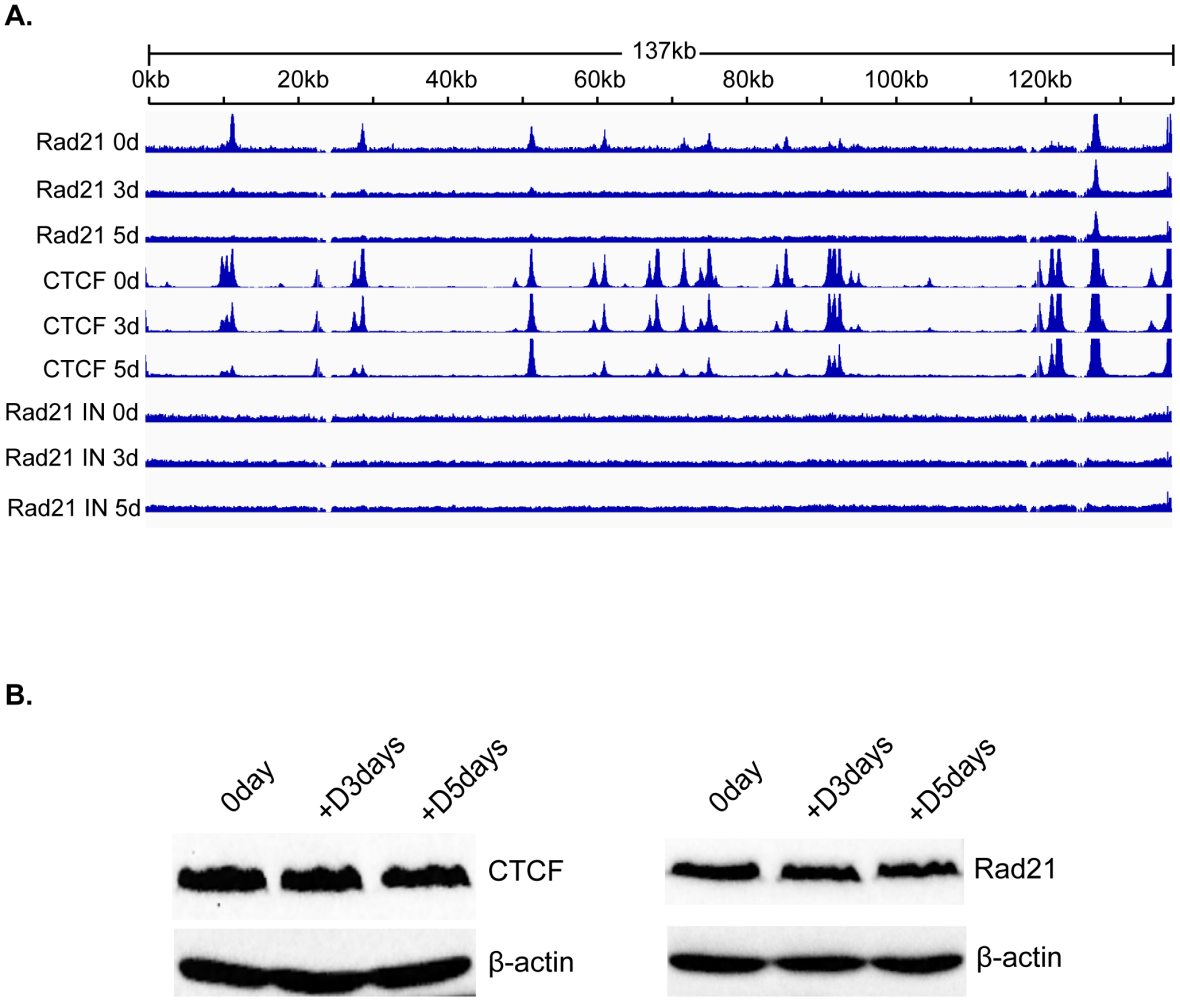
Changes in Rad21 binding to the KSHV genome during KSHV reactivation and lytic replication. A. ChIP-SEQ assays were performed on cell samples harvested at 0 d, 3 d, and 5 d after induction of lytic KSHV replication in iSLK cells. Relative read numbers are plotted on the vertical axis versus the reference KSHV genome map on the horizontal axis. Rad21 ChIP results from each time point are shown on the upper three panels and the corresponding CTCF ChIP results are shown on the middle three panels for comparison. The corresponding input samples (IN) are shown on the lower three panels. The tracks depict coverage per base, scaled per million mapped KSHV reads. B. CTCF and Rad21 levels in iSLK cells during KSHV replication. Protein lysates from iSLK cells at 72 and 120 hours after induction of lytic replication were immunoblotted with anti-CTCF or Rad21 antibodies. Blots were stripped and re-probed with anti-actin antibody as a loading control (lower panels).

Although cohesin is known to bind to many CTCF sites, the binding patterns of cohesin and CTCF to the human genome are not completely concordant [34]–[36]. Since Rad21 depletion appeared to have much more potent effects on KSHV lytic replication than CTCF depletion, it was critical to map the binding of Rad21 during lytic replication and compare its pattern to that of CTCF binding during the same period. We therefore performed a ChIP-seq analysis of Rad21 localization analogous to that conducted for CTCF. KSHV-infected iSLK cells were treated with doxycycline, and DNA was harvested for ChIP at 0, 3 and 5 days post-induction. Several significant differences between Rad21 and CTCF binding were immediately revealed by the ChIP-Seq analysis (Figure 5). Comparison with the previously described CTCF experiment demonstrates that there are twelve major peaks of Rad21 binding, significantly fewer than for CTCF. These are consistent with previously identified cohesin-binding sites but include at least one additional novel Rad21 binding locus at nt 28819–29553 (24,26). In addition, the profile of Rad21 differs from that of CTCF, with most peaks being much narrower, and the relative ratios of the major peaks differing from those of CTCF. Finally, and most interestingly, the eviction of Rad21 was much more rapid and generalized. Thus residual Rad21 binding at 72 h was only detectable at the two loci centered at approximately nucleotides 124,000 and 136,000, whereas the loss of CTCF binding was more gradual, and fully evident only by 5 days, in addition to being more site-specific. At 72 h, the input KSHV DNA copy number was only increased by 2.5 fold when measured by qPCR or estimated by viral read number in the ChIP-seq input samples, whereas Rad21 binding was absent at most sites. These data suggest that not only does Rad21 not bind to newly replicated genomes but that it is removed from pre-existing latent genomes.

To determine whether the changes in CTCF and Rad21 occupancy of the KSHV genome during lytic replication were associated with overall changes in the cellular levels of these proteins during KSHV reactivation, we performed immunoblotting of cell lysates harvested at serial time points when ChIP-Seq was performed. There was no detectable difference in the overall levels of either protein during the time period during which ChIP-seq was performed (Figure 5B).

### Depletion of host cell CTCF and Rad21 lead to distinct changes in the KSHV transcriptional profile during lytic replication

**Figure 6:**
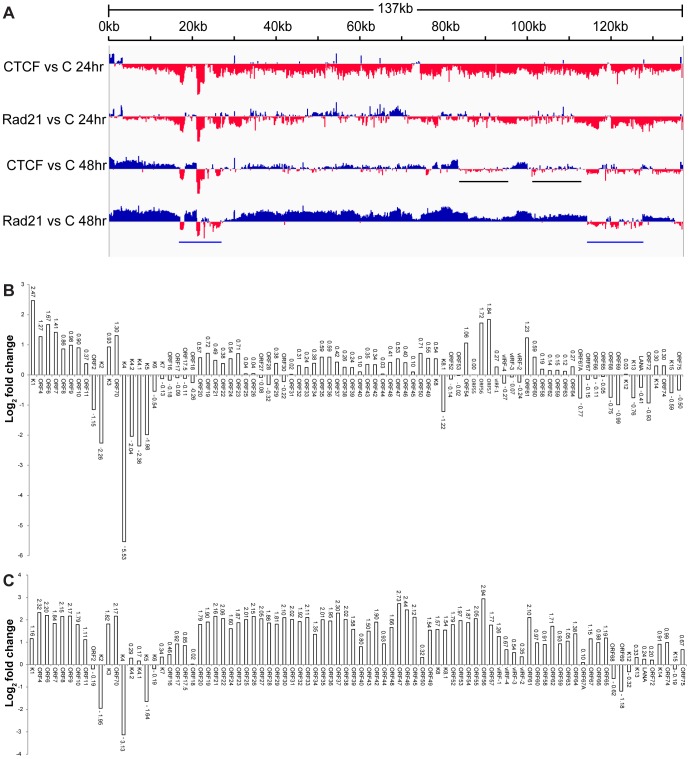
Effect of CTCF and Rad21 depletion on the KSHV lytic gene transcriptional profile defined by RNA-Seq. A. Transcriptome of iSLK cells at 24(C) are shown on the y-axis and the KSHV genome position on the x axis. Sites where CTCF or Rad21 knockdown leads to increased transcription compared to control are represented above the x-axis in blue. Regions where CTCF or Rad21 knockdown leads to decreased transcription compared to control are shown below the x-axis in red. Black bars show gene groups where the effects of CTCF and Rad21 differ and blue bars show genes that are CTCF and Rad21 dependent rather than repressed (see text). B. Effect of CTCF depletion on KSHV mRNAs. The effect of CTCF depletion on each annotated KSHV transcript is depicted as the log_2_ ratio of its RNA abundance in the absence versus presence of CTCF at 48 h after induction. Transcripts whose levels increase with CTCF knockdown are thus shown above the x-axis and transcripts that decrease in abundance with CTCF knockdown are shown below. C. Effect of Rad21 depletion on KSHV mRNAs. The effect of Rad21 depletion on each annotated KSHV transcript is depicted as the log_2_ ratio of its RNA abundance in the absence versus presence of Rad21 at 48 h after induction as described in panel (B) above.

The stimulatory effects of CTCF and Rad21 knockdown on KSHV production suggested that both proteins exert restrictive effects on KSHV lytic replication. Rad21 depletion led to significantly greater increases in KSHV yield, suggesting that Rad21 and CTCF might have unique effects on the transcription of KSHV lytic genes. In order to perform a comprehensive analysis and comparison of the effects of CTCF and Rad21 on the KSHV transcriptional profile, we performed high-throughput deep sequencing of mRNA from KSHV-infected cells in which either CTCF or Rad21 was depleted prior to induction of lytic replication. KSHV-infected iSLK cells were transfected with either control siRNA, CTCF siRNA or Rad21 siRNA as was done in the experiments to examine the effect on virion production. 48 hours after siRNA transfection, cells were treated with doxycycline to induce KSHV lytic replication, and cells were harvested at 24 and 48 hours after induction of replication. RNA was isolated, oligo-dT selected, and processed for deep sequencing. The effects of both CTCF and Rad21 knockdown on lytic cycle transcription were compared to each other and to the transcriptional profile of induced cells transfected with control siRNA. A comparison of the transcriptional profiles over time from each sample (control, CTCF-depleted and Rad21-depleted) is presented in Figure 6A, with the read counts normalized against the read counts in the induced control siRNA sample. The first somewhat surprising finding is that transcription of most KSHV genes actually decreases at 24 hours in the CTCF and Rad21 depleted cells compared to control. This is particularly evident in the CTCF-KD case, but is reduced overall by either CTCF-KD or Rad21-KD, suggesting that CTCF and Rad21 initially act as positive factors in lytic gene expression (Figure 6A and Figure S3). However, by 48 hours, lytic transcription of most genes is increased compared to control when CTCF is depleted. This biphasic effect on KSHV transcription was also evident upon Rad21 KD, with levels of the majority of lytic transcripts being increased by 48 hours. The ultimate enhancing effect of Rad21 on lytic gene transcription was even more pronounced than that of CTCF depletion, demonstrating that the two proteins have similar but distinct effects on lytic gene transcription.

In order to allow a more precise comparison, these data were analyzed by comparing read counts for each KSHV gene and the results are presented as binary comparisons between control versus CTCF KD and control versus Rad21 KD in Figures 6B and 6C, respectively. The net effect of CTCF on KSHV lytic gene expression is clearly repressive, as there was increased accumulation of virtually all lytic cycle gene transcripts by 48 hours when CTCF was depleted (Figure 6B). The effect of Rad21 depletion on the transcriptional profile at 48 hours was very similar to that of CTCF, with an increase in expression of most lytic cycle genes (Figure 6C). Consistent with its effect on virus production, the enhancement of gene expression due to Rad21 KD was significantly greater than the effect of CTCF KD for most genes. Whereas most mRNA levels were increased approximately 2–3 fold by CTCF KD, the increase was in the 4–8 fold range when Rad21 was knocked down (Figure 6B and Figure 6C). It should be noted that these increases in lytic mRNA levels due to CTCF KD and Rad21KD are superimposed on those observed as a consequence of induced lytic replication in NC SiRNA cells - which were several orders of magnitude (16-fold to 1000-fold) greater than in uninduced cells (Figure S4). When the effect of CTCF or Rad21 on early versus late lytic genes was compared, there was no significant difference overall based on the known temporal class of gene expression. The mean fold-change in early gene transcript levels was 3.3+/−2.1 S.D versus 4.5+/−1.2 S.D. for late genes.

Comparison of CTCF KD and Rad21 KD also demonstrates that these differences in the magnitude of the Rad21 versus CTCF effects were not uniform across the genome, i.e. there were specific individual differences in mRNA abundance due to CTCF KD versus Rad21 KD. This is most clearly evident in two such regions highlighted by black bars in Figure 6A. These regions, which include the vIRF genes and ORF65, ORF66, ORF67 and ORF67A, demonstrate decreased expression with CTCF KD and increased expression with Rad21 KD.

A third group of genes was also readily evident in the comparison of transcriptomes generated from CTCF-depleted and Rad21-depleted cells. This group consisted of genes whose abundance decreased with CTCF and Rad21 KD at 24 h and remained depressed compared to control at 48 h, suggesting that unlike the majority of genes, they are particularly dependent on CTCF and Rad21 for efficient expression. This group of genes in two clusters (denoted by blue bars, Figure 6A) includes K2,K4, K5, K6, K7, ORF68 and ORF69 (Figure 6B and Figure 6C).

**Figure 7:**
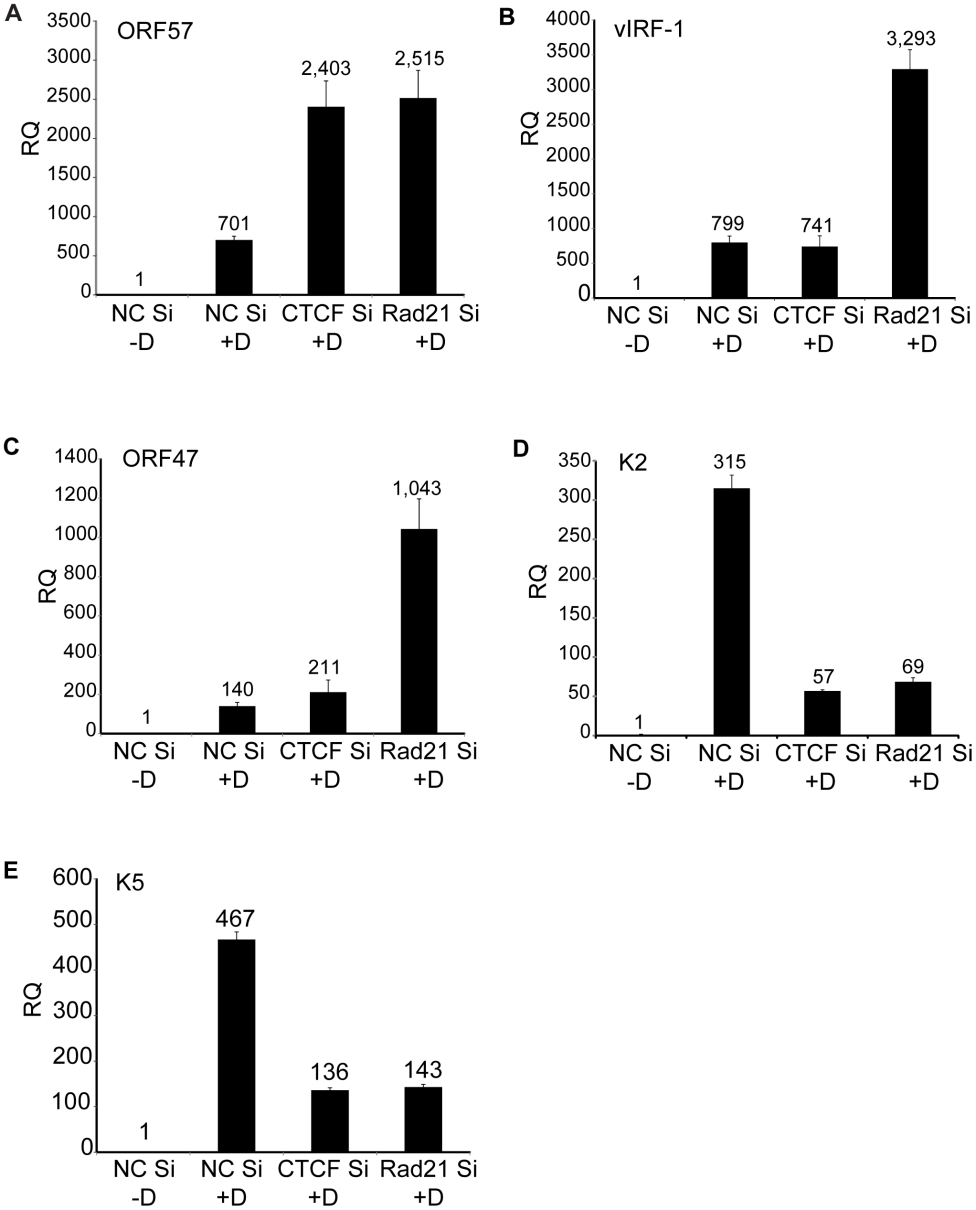
Effects of Rad21 or CTCF knockdown on expression of specific KSHV genes. CTCF or Rad21 knockdown was performed in iSLK cells with siRNAs specific for CTCF (CTCF Si) or Rad21 (Rad21 Si). Cells were also transfected with negative control siRNAs (NC Si). KSHV replication was induced with doxycycline (+D) or mock-induced (−D), and RNA was harvested 48 h after induction. qPCR was performed with primers specific for ORF57 (A), vIRF-1 (B), ORF47 (C), K2 (D) and K5 (E). Expression of each mRNA (RQ) was normalized to the level of expression in uninduced control cells (NC Si −D).

The magnitude of the overall changes in transcription of individual lytic genes due to CTCF or Rad21 knockdown were reproducible but relatively modest (2–8 fold over control) compared with the increases observed in virion production under the same conditions. At least 18 million reads were measured for each sample in the RNA-Seq analyses, which should allow accurate quantification of mRNA levels for all KSHV transcripts, which are abundantly expressed during lytic replication [37]. We therefore performed qPCR for selected mRNA targets to validate and confirm the RNASeq data. ORF57 is representative of the vast majority of genes whose expression was similarly upregulated by both CTCF and Rad21 KD (Figure 7A). The increase in ORF57 expression measured by qPCR upon CTCF or Rad21 KD was approximately 3.6 fold over control, which correlates well with the increases measured by RNASeq (4 fold). A second group of genes that were differentially regulated by CTCF and Rad21 is represented by vIRF1 and ORF47. Expression of both genes was not significantly changed by CTCF KD but was upregulated 5-fold by Rad21 KD (Figure 7B and 7C). The third group of genes, those whose expression was reduced by both CTCF KD and Rad21 KD, and are thus dependent on CTCF and Rad21 for expression, is represented by K2 and K5 (Figure 7D and 7E). Expression of both genes was confirmed to be reduced to 20% of control by both CTCF and Rad21 KD.

**Figure 8:**
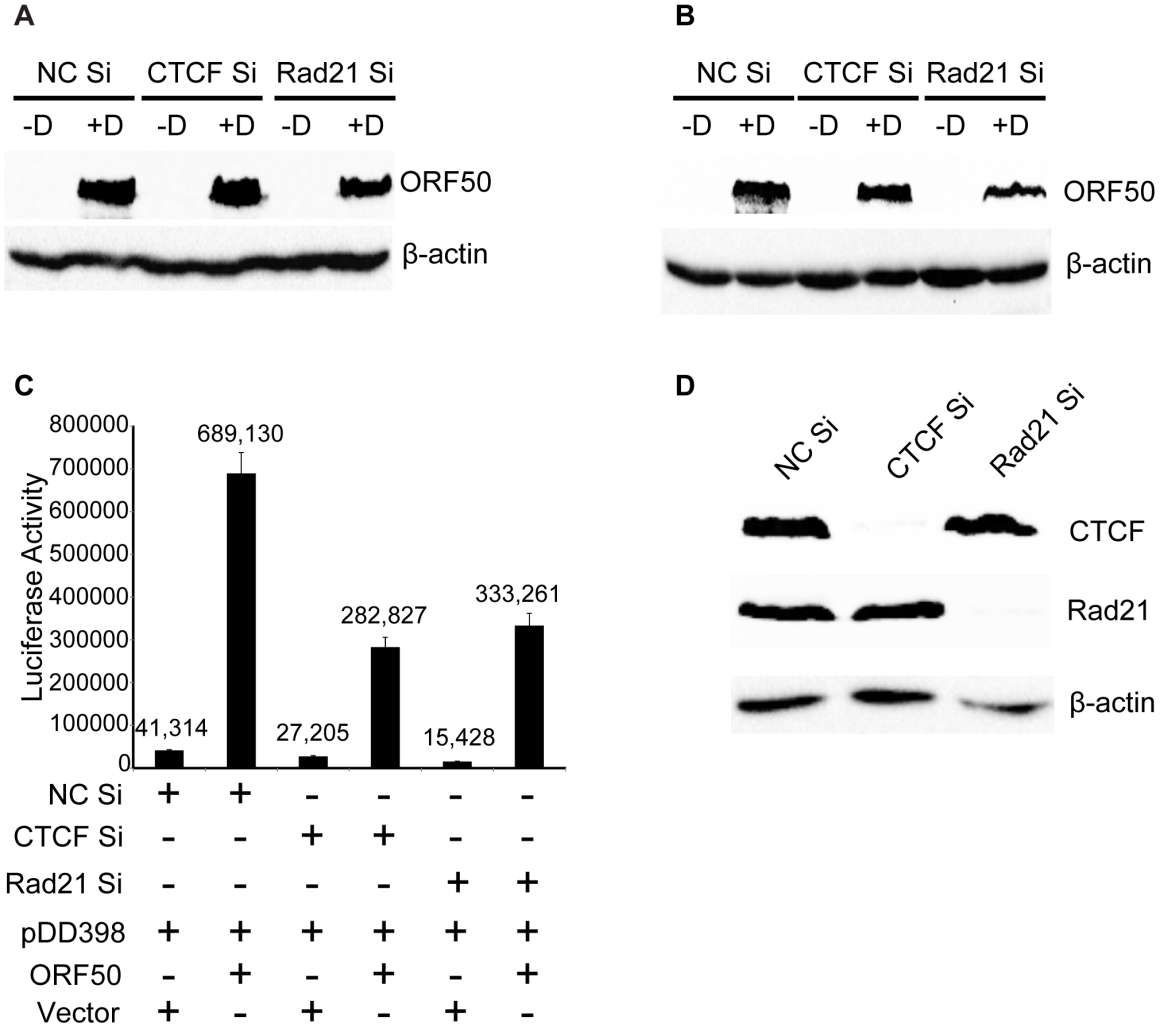
Lack of CTCF or Rad21 effect on ORF50/RTA levels or ORF50/RTA activation function. A. KSHV-infected iSLK cells were induced to replicate after control KD (NC Si), CTCF KD (CTCF Si) or Rad21 KD (Rad21 Si). Cells were either induced to permit KSHV replication with doxycycline (+D) or mock-induced (−D). Protein lysates were prepared at 24 hours post-induction and blotted with anti-ORF50/RTA antibodies. B. Samples were prepared from cells treated as in (A) above but harvested at 48 h post-induction and analyzed by immunoblotting with ORF50/RTA antibodies. C. Effect of CTCF or Rad21 depletion on ORF50/RTA transcriptional activation function. 293 cells were depleted of CTCF (CTCF Si) or Rad21 (Rad21 Si) by siRNA transfection or transfected with a negative control siRNA (NC Si). 48 h after siRNA transfection, cells were transfected with an ORF57 promoter-luciferase reporter plasmid (DD398) and either ORF50 expression plasmid (ORF50) or empty vector (vector). Luciferase assays were performed 48 h after transfection and are expressed as relative luminescence units. D. Lysates of cells used for luciferase assays in (C) above were immunoblotted with anti-CTCF or anti-Rad21 antibodies to confirm complete depletion.

Although induction of lytic KSHV replication in this system was dependent on expression of RTA/ORF50 from a transgene under the control of a tetracycline-responsive promoter, it was still possible that significant amounts of RTA/ORF50 protein were produced from endogenous KSHV transcripts. In order to determine whether ORF50 levels were altered by CTCF or Rad21 KD, and thereby responsible for some of the observed transcriptional changes, we directly measured ORF50 protein levels at the same time points at which RNA-Seq was performed. Immunoblotting of protein lysates from cells at 24 hrs and 48 hrs after induction of lytic replication revealed no significant increases in ORF50 protein levels when either CTCF or Rad21 depletion was carried out prior to induction (Figure 8A). In fact, a slight decrease in ORF50 protein was observed at 48 h in all cases. In order to address the possibility that CTCF or Rad21 might inhibit ORF50 function per se, we conducted the following experiment in which we asked whether CTCF or Rad21 KD affects the ability of ORF50 to activate RTA-responsive promoters in KSHV-negative cells. We performed luciferase assays with cells transfected with an RTA-responsive reporter plasmid and an RTA expression plasmid after either CTCF, Rad21 or control KD. The results shown in Figure 8C, demonstrate that CTCF and Rad21 do not inhibit RTA function in the reporter assay. Rather CTCF or Rad21 depletion actually resulted in slightly decreased RTA activation function. These results together demonstrate that the global effects of CTCF and Rad21 on KSHV lytic gene expression are not likely to be mediated via effects on RTA expression or function.

## Discussion

In this study we report several novel aspects of the role of CTCF and cohesin as regulators of KSHV virus production. First, both CTCF and Rad21 act as host restriction factors for lytic KSHV replication as depletion of either protein resulted in markedly increased production of infectious virions. Rad21 appears to exert a greater effect, as Rad21 knockdown resulted in nearly 100-fold increases in virus yield, approximately five times more than the increase caused by CTCF knockdown. We also demonstrate that both CTCF and Rad21 dissociate from viral genomes during the process of lytic KSHV replication. Rad21 binding is lost earlier and more completely than CTCF after lytic KSHV replication begins. The almost complete loss of Rad21 from the majority of KSHV genome sites indicates eviction from latent episomes early during lytic replication as well as a lack of binding to newly replicated genomes. Conversely, the persistence of Rad21 at the major latency region and the terminal repeats indicates that Rad21 not only remains bound to template genomes but that it binds to nascently replicated genomes at these two sites. CTCF also exhibited site-specific changes in KSHV genome occupancy during lytic replication. CTCF occupancy was decreased by 3 days, and by 5 days, the relative occupancy at most sites was reduced by over 50%, indicating that CTCF binding also does not occur to newly replicated genomes at these locations. The finding that CTCF depletion results in increased virus production is in contrast to those of Chen et. al. who did not observe any effects of CTCF knockdown on KSHV lytic transcription in PEL cells [24]. These differences may be due to the different cell lines employed, and to the fact that knockdown in the our experiments was essentially complete, with no detectable CTCF remaining at the time of lytic induction.

Our findings that cohesin and CTCF may play distinct roles in regulating KSHV reactivation and virion production are mirrored by the differing effects of their knockdown on KSHV lytic gene transcription. Consistent with the more profound effects of Rad21 KD on virion production, Rad 21 depletion consistently led to greater increases in KSHV lytic gene expression than did CTCF KD. Further, depletion of the two proteins had distinguishable effects on the lytic transcriptional profile. Whereas Rad21 KD led to increases in several vIRF gene transcript levels, compared to control, CTCF KD led to decreases or no change in this subset of mRNAs. A similar pattern was observed in several other specific genes, highlighting the complexity of overlapping gene regulation by CTCF and cohesin.

Another novel finding in our study is the kinetic profile of the effects of CTCF and Rad21 on lytic gene transcription. Upon induction of KSHV replication, lytic gene transcription increased several orders of magnitude at 24 hours, as expected, and increased further at 48 hours. When CTCF or Rad21 were depleted, the increases in lytic gene transcription were significantly depressed at 24 hours compared to control. This relative decrease in lytic transcription was reversed by 48 hours, when CTCF KD, and particularly Rad21 KD, resulted in greater accumulation of lytic transcripts than in the presence of either protein. These data suggest that at baseline, CTCF and cohesin act as stimulators of transcription of many KSHV lytic genes but that their net effect, exerted subsequently, is negative, resulting in overall restriction of transcription and virus production. What is the likely mechanism of cohesin and CTCF gene activation, followed by inhibition? It has been suggested that cohesin binding to the promoter region of ORF50/RTA and secondary interactions with cohesin bound at the latency promoter have a repressive effect on ORF50 expression, which is required for lytic reactivation, thus acting as a proximal inhibitor of lytic transcription [24]. Our data suggest that the effects of cohesin on KSHV lytic gene transcription are more complex and global. First, lytic replication was initiated by expression of RTA *in trans*, essentially removing RTA as a limiting factor for lytic transcription. In addition, total levels of RTA protein were not affected by CTCF or Rad21 depletion. Finally, depletion of cohesin and CTCF actually resulted in less accumulation of KSHV transcripts at early times. These data are consistent with cohesin and CTCF initially acting as general stimulators of KSHV lytic transcription, similar to cohesin's effect on host cell genes [23], [38]. Cohesin appears to stimulate transcription from promoters of genes to which it is bound by facilitating the transition from paused RNA polII to elongating polII [23], [39]. In addition, cohesin increases polII occupancy at genes to which it binds, most likely by increasing enhancer-promoter contact via looping. Importantly, however, depletion of cohesin also decreases transcription at most genes which do not bind cohesin and do not contain paused promoters, likely due to cohesin effects on basal and specific transcription factors [23], [40]. It is this latter mechanism which is most likely responsible for the globally decreased KSHV transcription seen at earlier times after cohesin depletion in our studies. The mechanisms by which CTCF and cohesin regulate herpesvirus transcription are likely to be significantly different from those operative on the human genome as herpesvirus lytic genes are virtually all unspliced and in close proximity to each other. Thus facilitation of enhancer-promoter interaction may be less important in regulation of herpesvirus transcription by cohesin. Combined with the relatively limited number of high density cohesin sites on the KSHV genome, the positive effects of cohesin on early lytic gene transcription are likely due to the indirect effects of cohesin on cellular transcription factors referred to above.

What is the likely basis of the subsequent enhancing effects of cohesin depletion on lytic gene expression and RNA accumulation? A possible mechanism is suggested by the requirement for DNA replication *in cis* for efficient transcription of late lytic herpesvirus promoters. The positive effects of DNA replication *in cis* on transcription may derive from topological changes facilitating access to transcription factors as well as relocalization of genomes to intranuclear replication compartments [41]–[43]. It is possible that the physical linkages between cohesin molecules at various sites on circular latent genomes constrain the molecule, limiting maximal transcription. The more robust effect of Rad21 depletion on transcription, and especially virus production, suggest that the linking effects of cohesin may be more important in this regard than CTCF. It also implies that cohesin binding, although coincident with CTCF, may not be completely abrogated by removal of CTCF. The distinct and separable nature of cohesin and CTCF functions is underscored by the subtle but clear differences in the KSHV transcriptional profile exerted by their individual depletion.

An additional insight into the potential role of cohesin in regulating KSHV transcription is provided by an examination of the few genes whose transcript levels are depressed by cohesin depletion and remain suppressed at later times. These include several of the K transcripts, suggesting that they are particularly dependent on cohesin for their efficient expression. It is likely relevant that several of these same genes were previously identified as unique among KSHV genes in containing paused RNA polII at their promoters [44]. It has recently been demonstrated that cohesin is particularly important for transcription of eukaryotic promoters that contain paused RNA polII. Thus cohesin may play the same role at these particular KSHV promoters as it does at a subset of cellular promoters that contain paused RNA polII, facilitating transition to elongation [23]. Whether such pausing also occurs at the other KSHV genes whose expression is adversely affected by cohesin depletion (e.g. ORF 68, ORF69) or if there are other promoter properties that determine cohesin dependence is an interesting avenue for further study.

In summary, CTCF and cohesin play distinct roles in regulating KSHV reactivation from latency at the level of mRNA transcription. Cohesin and CTCF appear to initially act as positive factors, facilitating transcription for the majority of KSHV lytic genes, but subsequently their presence limits transcription and virus production, potentially by topological effects on transcription. In contrast to its role in host cell gene regulation, cohesin may primarily play an inhibitory role in transcriptional control of the KSHV lytic cycle. With regards to its baseline stimulatory effects on transcription in KSHV, cohesin effects may primarily derive from global effects on transcription factors such as myc, as has been previously demonstrated with cellular promoters [23], [40]. It is less likely that cohesin stimulates transcription by facilitating long-range enhancer recruitment to specific promoters as observed in cellular eukaryotic systems [22], [23]. In addition, cohesin and CTCF appear to be required for activity of certain KSHV promoters that are particularly cohesin and CTCF-dependent, and these effects are possibly due to effects on paused RNA polII. During the process of KSHV virion production, cohesin, and to a lesser degree, CTCF, dissociate from latent KSHV genomes, implying a dynamic role for both in replication control. The importance of both proteins as host restriction factors regulating KSHV reactivation is demonstrated by the dramatic increases in virus yield that result from their depletion.

## Supporting Information

Figure S1 **KSHV virus production in cells depleted of Rad21 or CTCF with additional siRNA pools and negative control siRNAs.** A. CTCF knockdown (CTCF SiG) and Rad21 knockdown (Rad21 SiG) were performed on iSLK cells in parallel with control siRNA transfection (NC Si#5). KSHV replication was induced by addition of doxycycline (+D). Supernatants from induced cells were used to infect 293 cells. Virus passage was quantitated by flow cytometry of GFP-positive 293 cells. Each transfection/induction was performed in triplicate and three replicate infections were performed with each supernatant. No virus was detected in uninduced virus supernatants (data not shown). B. Immunoblotting of lysates from cells used in virus production experiments in panel A above was performed with anti-CTCF and anti-Rad21 antibodies to verify completeness of CTCF and Rad21 depletion. Lysates were prepared from cells harvested at the time of replication induction with doxycycline.
(TIF)

Figure S2 **Effect of siRNAs on virus passage measured by GFP transduction.** A standard virus passage assay was performed by incubation of 293 cells with diluted KSHV-containing supernatant (NC Si +D sup). To parallel infections, 5 ul of supernatant from siRNA-transfected cells (but not induced) was added. Supernatant from cells transfected with either CTCF-specific siRNA (CTCF Si) or Rad21-specific siRNA (Rad21 Si) was added. All infections were performed in triplicate and flow cytometry determinations were performed in triplicate.
(TIF)

Figure S3 **Effect of CTCF and Rad21 depletion on the KSHV lytic gene transcriptional profile defined by RNA-Seq at 24 h.** A. Effect of CTCF depletion on KSHV mRNAs. The effect of CTCF depletion on each annotated KSHV transcript is depicted as the log_2_ ratio of its RNA abundance in the absence versus presence of CTCF at 24 h after induction. Transcripts whose levels increase with CTCF knockdown are thus shown above the x-axis and transcripts that decrease in abundance with CTCF knockdown are shown below. B. Effect of Rad21 depletion on KSHV mRNAs. The effect of Rad21 depletion on each annotated KSHV transcript is depicted as the log_2_ ratio of its RNA abundance in the absence versus presence of Rad21 at 24 h after induction as described in panel (A) above.
(TIF)

Figure S4 **Comparison of gene expression in cells induced to permit KSHV lytic replication versus uninduced cells.** KSHV-infected iSLK cells were treated with doxycycline to induce KSHV replication or mock-treated to serve as the uninduced control. RNA was harvested at 24 h and 48 h from each sample and RNA sequencing was performed. A. Transcriptome of iSLK cells at 24 h and 48 h after induction of lytic replication. The log ratios of read number for each position on the KSHV genome in cells induced to permit replication (In) versus the values for corresponding uninduced control cells (Un) are shown on the y-axis and the KSHV genome position on the x axis. Sites where replication leads to increased transcription compared to control are represented above the x-axis in blue. Sites where replication leads to decreased transcription compared to control are shown below the x-axis in red. B. Effect of replication on lytic gene expression at 24 h. The log_2_ ratio of the RNA abundance for each gene in replication-induced cells versus uninduced cells at 24 h after induction is shown on the y-axis and the gene name is shown on the x-axis. Transcripts whose levels increase with KSHV replication are thus shown above the x-axis and transcripts that decrease in abundance are shown below. C. Effect of replication on lytic gene expression at 48 h. Changes in KSHV transcript abundance at 48 h after induction of replication for each gene are expressed as log_2_ ratios of mRNA levels in induced versus uninduced cells, as in (B) above.
(TIF)
