# Supplementary figures and images for: CTCF and Rad21 Act as Host Cell Restriction Factors for Kaposi's Sarcoma-Associated Herpesvirus (KSHV) Lytic Replication by Modulating Viral Gene Transcription
Source: PLoS Pathog. 2014 Jan 9;10(1):e1003880. doi: 10.1371/journal.ppat.1003880 (PMC3887114; doi:10.1371/journal.ppat.1003880)

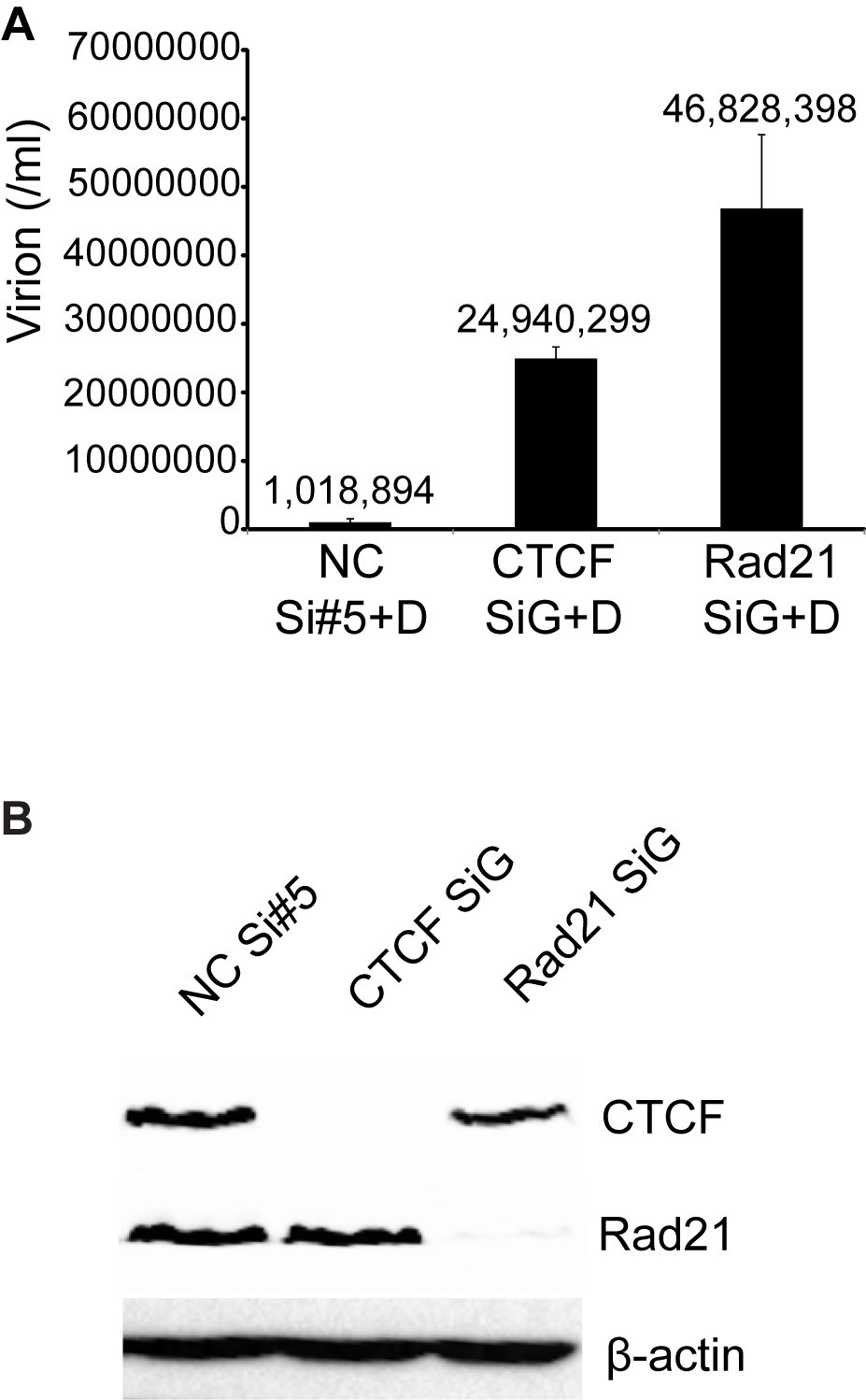

Supplement: Figure S1 — KSHV virus production in cells depleted of Rad21 or CTCF with additional siRNA pools and negative control siRNAs. A. CTCF knockdown (CTCF SiG) and Rad21 knockdown (Rad21 SiG) were performed on iSLK cells in parallel with control siRNA transfection (NC Si#5). KSHV replication was induced by addition of doxycycline (+D). Supernatants from induced cells were used to infect 293 cells. Virus passage was quantitated by flow cytometry of GFP-positive 293 cells. Each transfection/induction was performed in triplicate and three replicate infections were performed with each supernatant. No virus was detected in uninduced virus supernatants (data not shown). B. Immunoblotting of lysates from cells used in virus production experiments in panel A above was performed with anti-CTCF and anti-Rad21 antibodies to verify completeness of CTCF and Rad21 depletion. Lysates were prepared from cells harvested at the time of replication induction with doxycycline. (TIF) [file ppat.1003880.s001.tif]

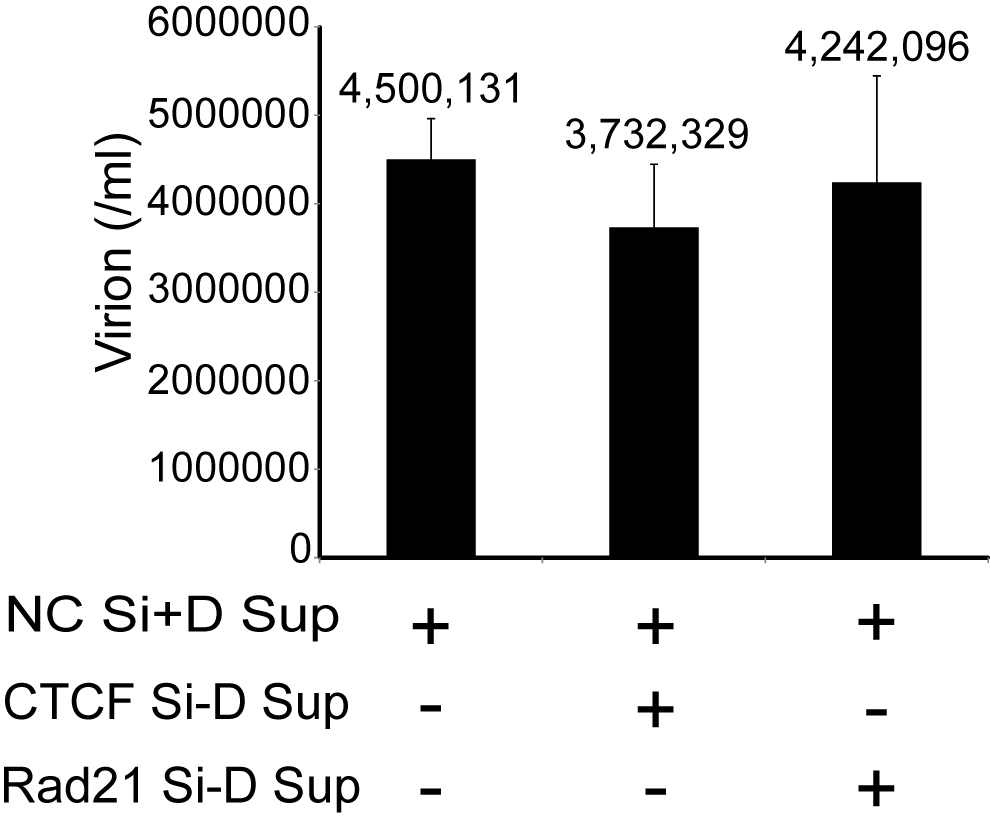

Supplement: Figure S2 — Effect of siRNAs on virus passage measured by GFP transduction. A standard virus passage assay was performed by incubation of 293 cells with diluted KSHV-containing supernatant (NC Si +D sup). To parallel infections, 5 ul of supernatant from siRNA-transfected cells (but not induced) was added. Supernatant from cells transfected with either CTCF-specific siRNA (CTCF Si) or Rad21-specific siRNA (Rad21 Si) was added. All infections were performed in triplicate and flow cytometry determinations were performed in triplicate. (TIF) [file ppat.1003880.s002.tif]

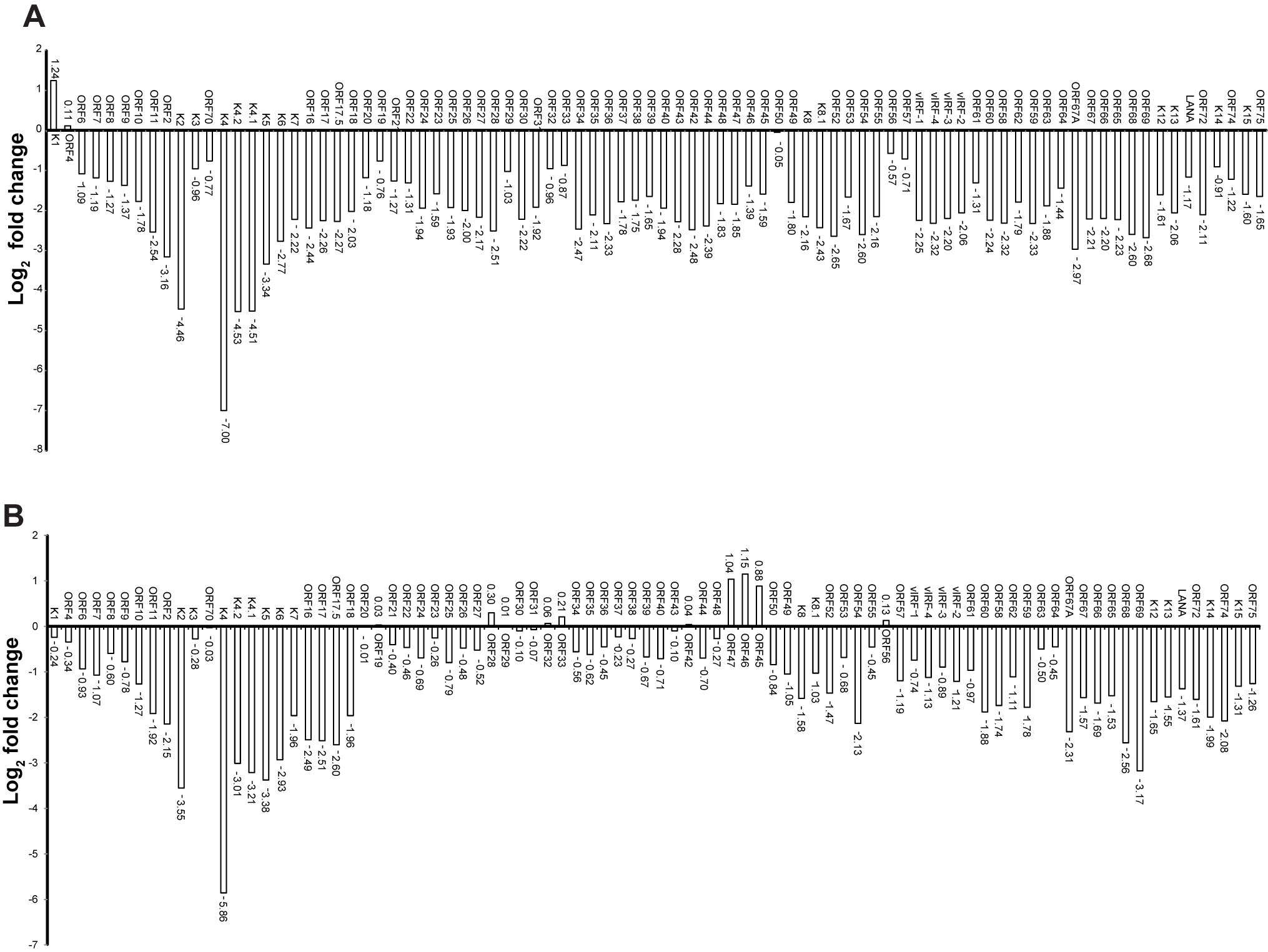

Supplement: Figure S3 — Effect of CTCF and Rad21 depletion on the KSHV lytic gene transcriptional profile defined by RNA-Seq at 24 h. A. Effect of CTCF depletion on KSHV mRNAs. The effect of CTCF depletion on each annotated KSHV transcript is depicted as the log2 ratio of its RNA abundance in the absence versus presence of CTCF at 24 h after induction. Transcripts whose levels increase with CTCF knockdown are thus shown above the x-axis and transcripts that decrease in abundance with CTCF knockdown are shown below. B. Effect of Rad21 depletion on KSHV mRNAs. The effect of Rad21 depletion on each annotated KSHV transcript is depicted as the log2 ratio of its RNA abundance in the absence versus presence of Rad21 at 24 h after induction as described in panel (A) above. (TIF) [file ppat.1003880.s003.tif]

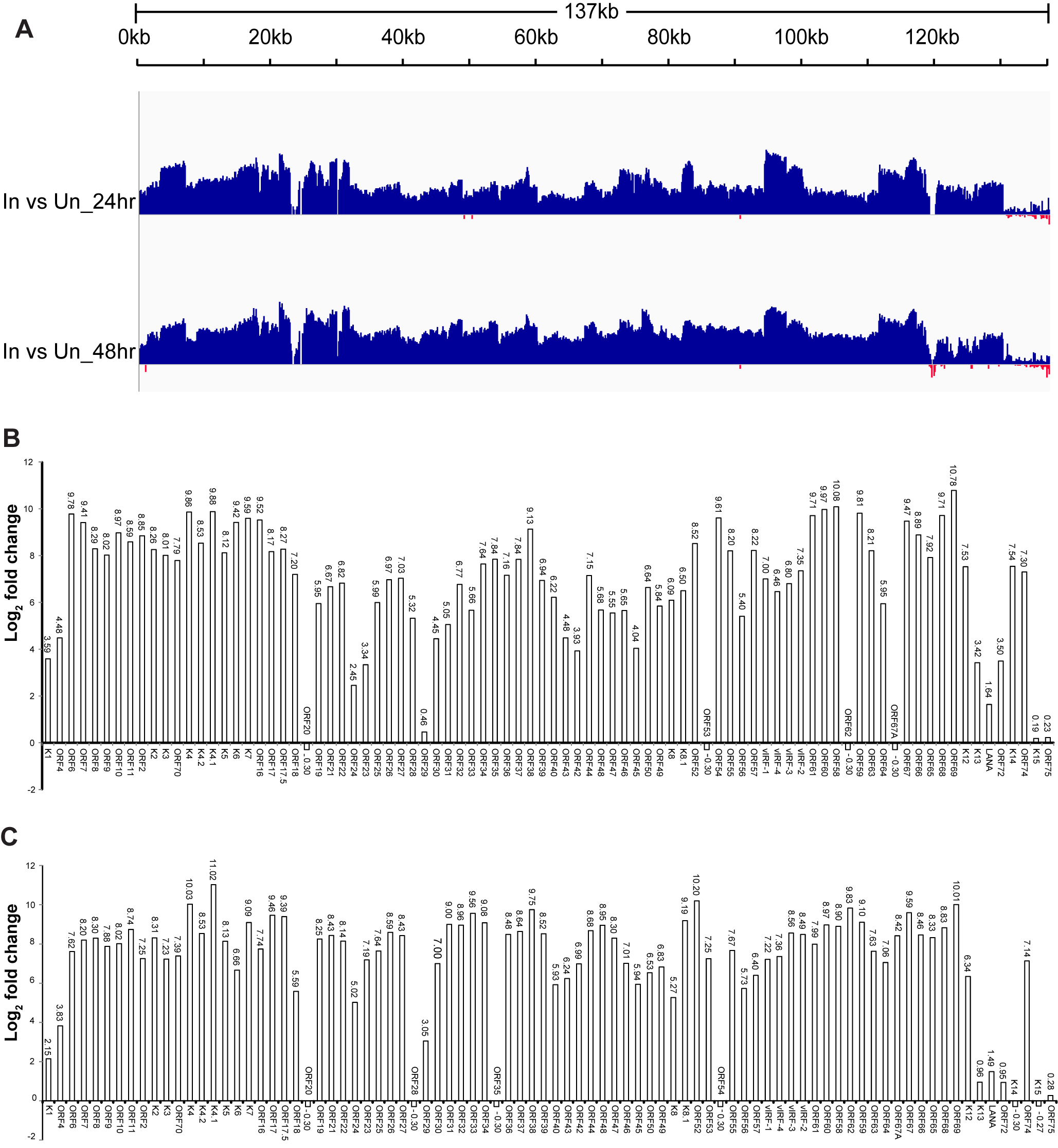

Supplement: Figure S4 — Comparison of gene expression in cells induced to permit KSHV lytic replication versus uninduced cells. KSHV-infected iSLK cells were treated with doxycycline to induce KSHV replication or mock-treated to serve as the uninduced control. RNA was harvested at 24 h and 48 h from each sample and RNA sequencing was performed. A. Transcriptome of iSLK cells at 24 h and 48 h after induction of lytic replication. The log ratios of read number for each position on the KSHV genome in cells induced to permit replication (In) versus the values for corresponding uninduced control cells (Un) are shown on the y-axis and the KSHV genome position on the x axis. Sites where replication leads to increased transcription compared to control are represented above the x-axis in blue. Sites where replication leads to decreased transcription compared to control are shown below the x-axis in red. B. Effect of replication on lytic gene expression at 24 h. The log2 ratio of the RNA abundance for each gene in replication-induced cells versus uninduced cells at 24 h after induction is shown on the y-axis and the gene name is shown on the x-axis. Transcripts whose levels increase with KSHV replication are thus shown above the x-axis and transcripts that decrease in abundance are shown below. C. Effect of replication on lytic gene expression at 48 h. Changes in KSHV transcript abundance at 48 h after induction of replication for each gene are expressed as log2 ratios of mRNA levels in induced versus uninduced cells, as in (B) above. (TIF) [file ppat.1003880.s004.tif]
